# Participation of Lower and Upper Middle–Income Countries in Clinical Trials Led by High-Income Countries

**DOI:** 10.1001/jamanetworkopen.2022.27252

**Published:** 2022-08-18

**Authors:** Fidel Rubagumya, Wilma M. Hopman, Bishal Gyawali, Deborah Mukherji, Nazik Hammad, C. S. Pramesh, Mykola Zubaryev, Alexandru Eniu, Audrey T. Tsunoda, Tezer Kutluk, Ajay Aggarwal, Richard Sullivan, Christopher M. Booth

**Affiliations:** 1 Department of Clinical Oncology, Rwanda Military Hospital, Kigali, Rwanda; 2 Division of Cancer Care and Epidemiology, Queen’s University Cancer Research Institute, Kingston, Ontario, Canada; 3 Department of Oncology, Queen’s University, Kingston, Ontario, Canada; 4 Public Health Sciences, Queen’s University, Kingston, Ontario, Canada; 5 Department of Oncology, American University of Beirut Medical Center, Beirut, Lebanon; 6 Department of Oncology, Tata Memorial Centre, Homi Bhabha National Institute, Mumbai, India; 7 Department of Surgical Oncology, National Cancer Institute of Ukraine, Kyiv, Ukraine; 8 Department of Oncology, Hopital Riviera-Chablais, Rennaz, Switzerland; 9 Department of Oncology, Hospital Erasto Gaertner e PUCPR, Curitiba, Paraná, Brazil; 10 Department of Oncology, Hacettepe University Faculty of Medicine & Cancer Institute, Ankara, Turkey; 11 Institute of Cancer Policy, King’s College London, London, United Kingdom; 12 London School of Hygiene and Tropical Medicine, London, United Kingdom; 13 Institute of Cancer Policy, King's College London, London, United Kingdom

## Abstract

**Importance:**

Many randomized clinical trials (RCTs) led by high-income countries (HICs) now enroll patients from lower middle–income countries (LMICs) and upper middle–income countries (UMICs). Although enrolling diverse populations promotes research collaborations, there are issues regarding which countries participate in RCTs and how this participation may contribute to global research.

**Objective:**

To describe which UMICs and LMICs participate in RCTs led by HICs.

**Design, Setting, and Participants:**

A cross-sectional study of all oncology RCTs published globally during January 1, 2014, to December 31, 2017, was conducted. The study cohort was restricted to RCTs led by HICs that enrolled participants from LMICs and UMICs. Study analyses were conducted in November 1, 2021, to May 31, 2022.

**Main Outcomes and Measures:**

A bibliometric approach (Web of Science 2007-2017) was used to explore whether RCT participation was proportional to other measures of cancer research activity. Participation in RCTs (ie, percentage of RCTs in the cohort in which each LMIC and UMIC participated) was compared with country-level cancer research bibliometric output (ie, percentage of total cancer research bibliometric output from the same group of countries that came from a specific LMIC and UMIC).

**Results:**

Among the 636 HIC-led RCTs, 186 trials (29%) enrolled patients in LMICs (n = 84 trials involving 11 LMICs) and/or UMICs (n = 181 trials involving 26 UMICs). The most common participating LMICs were India (42 [50%]), Ukraine (39 [46%]), Philippines (23 [27%]), and Egypt (12 [14%]). The most common participating UMICs were Russia (115 [64%]), Brazil (94 [52%]), Romania (62 [34%]), China (56 [31%]), Mexico (56 [31%]), and South Africa (54 [30%]). Several LMICs are overrepresented in the cohort of RCTs based on proportional cancer research bibliometric output: Ukraine (46% of RCTs but 2% of cancer research bibliometric output), Philippines (27% RCTs, 1% output), and Georgia (8% RCTs, 0.2% output). Overrepresented UMICs include Russia (64% RCTs, 2% output), Romania (34% RCTs, 2% output), Mexico (31% RCTs, 2% output), and South Africa (30% RCTs, 1% output).

**Conclusions and Relevance:**

In this cross-sectional study, a substantial proportion of RCTs led by HICs enrolled patients in LMICs and UMICs. The LMICs and UMICs that participated in these trials did not match overall cancer bibliometric output as a surrogate for research ecosystem maturity. Reasons for this apparent discordance and how these data may inform future capacity-strengthening activities require further study.

## Key Points

**Question:**

Which upper middle–income countries (UMICs) and lower middle–income countries (LMICs) participate in oncology randomized clinical trials led by high-income countries?

**Findings:**

In this cross-sectional study, among all 636 oncology randomized clinical trials published globally during 2014 to 2017, the most common participating LMICs were India (50% of trials), Ukraine (46%), and Philippines (27%). The most common participating UMICs were Russia (64% of trials), Brazil (52%), Romania (34%), China (31%), Mexico (31%), and South Africa (30%).

**Meaning:**

The findings of this study suggest LMICs and UMICs that participate in randomized clinical trials do not match overall cancer bibliometric output as a surrogate for research ecosystem maturity; reasons for this apparent discordance and how these data may inform future capacity-strengthening activities require further study.

## Teaser

This cross-sectional study examines the inclusion of patients with cancer from lower and upper middle–income countries in randomized clinical trials conducted by high-income countries.

## Introduction

Over the past 2 decades, there has been a marked increase in globalization of randomized clinical trials (RCTs); many RCTs led by high-income countries (HICs) are now enrolling patients from upper middle–income countries (UMICs) and lower middle–income countries (LMICs).^1,2^ Although enrolling diverse global populations promotes research collaborations and may improve access to novel therapies, ethical concerns have arisen regarding industry-led clinical trials in lower-resource settings. Several issues remain, including the population that benefits from the globalization of RCTs, potential exploitation of research participants in settings with less regulatory oversight, and whether novel therapeutics will be available in participating countries following completion of the RCTs.^3,4^

The Declaration of Helsinki stipulates that RCT teams make provisions for posttrial access to study medicine for all participants.^5^ However, there are reasons to be concerned that new medicines that are found to be efficacious in RCTs may not be widely available to patients in resource-constrained health systems that participate in these pivotal trials. Despite these risks, there are potential benefits beyond immediate drug access for patients enrolled in the RCTs; UMICs and LMICs that participate in global RCTs may benefit by building capacity, making them better positioned to lead their own trials in the future.^6^

There are concerns that recent trends in globalization of RCTs may be primarily associated with logistical considerations including the need for a large sample size to identify a small effect. In addition, there may be less oversight, lower costs, and fewer regulatory hurdles, which make lower-resource countries appealing to industry.^6,7^

Transparency in the conduct of global RCTs is highly variable; little is known about standards that govern enrollment of patients in UMICs and LMICs.^3,8^ There are potential complex ethical issues in these relationships. Trials led by industry may offer strong financial incentives for investigators and centers in UMICs and LMICs to participate, which may introduce a power dynamic that could facilitate RCTs and practices moving forward without the expected level of oversight.

Our group recently reported an overview of the design, conduct, and results of all cancer RCTs published globally during 2014 to 2017.^9^ In that overview we found that only 8% of oncology RCTs (58 of 694) were led by UMICs or LMICs; China (42 of 58 [72%]) and India (6 of 58 [10%]) accounted for most of these trials. The objective of the present study was to describe which UMICs and LMICs are contributing to RCTs led by HICs and examine whether this level of contribution reflects proportional national strengths in cancer research, using a bibliometric comparative analysis.

## Methods

### Study Design and Search Strategy

As described elsewhere,^9^ we have created a database of all oncology RCTs conducted globally during January 1, 2014, to December 31, 2017. We report methods and results in accordance with the Strengthening the Reporting of Observational Studies in Epidemiology (STROBE) reporting guideline. Phase 3 RCTs were included if they tested anticancer systemic therapy, radiotherapy, or surgery. Trials were excluded if they did not report the overall study primary end point. The present report represents a secondary analysis to understand the level of UMIC and LMIC involvement in the global research ecosystem. Accordingly, the study population was restricted to RCTs led by HICs that enrolled participants from UMICs and LMICs. This study made use of publicly reported data from published reports of RCTs; the Queen’s University Health Sciences and Affiliated Teaching Hospitals Research Ethics Board Institutional Review Board granted a waiver of informed consent.

### Data Abstraction and Outcomes

All eligible studies were reviewed using a data abstraction form to capture information regarding participant enrollment, funding, study design, and results. Data abstraction was performed independently by 2 members of the primary research team. The senior author (C.M.B.) performed random duplicate abstraction to ensure data abstraction was of high quality. At completion of data collection, 30 studies were randomly chosen for review; only 11 of 1020 variables (1%) were found to be discordant with the original assessment. Studies were classified into country of origin based on the institutional affiliation of the first author.

We used a bibliometric approach to explore whether the participation of UMICs and LMICs in RCTs led by HICs was proportional to a surrogate measure of cancer research activity (ie, bibliometric output) (eFigure 1 in the Supplement). Fractional counts were assigned for each author of the publications reviewed on the basis of their country address. For example, 4 authors from 4 countries on 1 article would each count for 0.25. Country-level bibliometric output for 2007 to 2017 was identified in the Web of Science database. We compared RCT participation (ie, percentage of RCTs in our cohort in which each LMIC and UMIC participated) with country-level cancer research bibliometric output (ie, percentage of total cancer research bibliometric output from the same group of countries that came from a specific LMIC and UMIC). Total cancer bibliometric output included all types of cancer research (eg, basic science, clinical research, and health system research). This process was done separately for 84 trials involving 11 LMICs and 181 trials involving 26 UMICs.

### Statistical Analysis

Descriptive results (frequencies and percentages for categorical data and medians and IQRs for continuous data) were generated using IBM SPSS, version 27.0 for Windows (IBM Corp). Comparisons were made between studies that were led by HICs and enrolled participants from UMICs and LMICs and those led by HICs with no UMIC or LMIC enrollment, using χ^2^ and Fisher exact tests for categorical data and the Mann-Whitney test for continuous data. We characterized trial differences between the 2 groups based on funding, types of cancers studied, types of experimental interventions, trial design, and outcomes. Journal impact factor was also compared using the impact factor from 2016 (regardless of year of publication), as reported by the journal citation reports impact factor.^10^ We also compared the effect size (as measured through hazard ratio) of positive superiority in RCTs between groups. All superiority studies of systemic therapy that met their primary end point were assigned a European Society of Medical Oncology–Magnitude of Clinical Benefit Results Scale grade.^11^ Findings were considered significant at *P* < .05 with 2-sided, unpaired testing; no adjustments for multiple comparisons were made.

## Results

### Results of the Search Strategy

The original search strategy identified 2275 publications. As shown in eFigure 2 in the Supplement, 1639 studies (72%) were not eligible for the present analysis. The global study cohort included 694 RCTs; 636 (92%) of these were led by HICs. Of 58 RCTs (8%) led by UMICs and LMICs, most were conducted by China (42 [72%]) and India (6 [10%]). Other UMICs and LMICs that led RCTs were Cuba (3 [5%]), Colombia, Egypt, Iran, Romania, and Serbia (1 each [2%]). Among the HIC-led trials, 186 (29%) enrolled patients in UMICs and LMICs. Of the 636 RCTs led by HICs, 84 (13%) enrolled patients from 11 LMICs and 181 (28%) enrolled patients from 26 UMICs; these groups formed the study cohort. Comparative analyses were performed with the 450 RCTs (65%) that only enrolled patients in HICs.

### Design Characteristics of RCTs

Among 84 HIC-led RCTs that involved 11 LMICs, the most common participating countries were India (42 [50%]), Ukraine (39 [46%]), Philippines (23 [27%]), and Egypt (12 [14%]). Among 181 HIC-led RCTs that involved 26 UMICs, the most common participating countries were Russia (115 [64%]), Brazil (94 [52%]), Romania (62 [34%]), China (56 [31%]), Mexico (56 [31%]), and South Africa (54 [30%]).

Characteristics of the study cohort are presented in Table 1. Of 186 RCTs that enrolled individuals from LCMIs and UMICs, the most common cancers studied were breast (38 [20%]), hematologic (36 [19%], lung (28 [15%]), gastrointestinal (25 [13%]), and urologic (24 [13%]). The distribution was similar for 450 HIC trials that did not include LMICs or UMICs (eg, breast, 77 [17%] and hematologic, 83 [18%]) (*P* = .18). The extent to which cancers studied in the 186 RCTs enrolling participants in LMICs and UMICs align with cancer mortality in the same settings is presented in Table 2. The proportion of oncology RCTs enrolling participants in LMICs and UMICs relative to cancer mortality in the same settings was relatively higher for breast (20% RCTs, 7% deaths), hematologic (19% RCTs, 7% deaths), and urologic (13% RCTs, 7% deaths) cancers. The proportion of RCTs compared with cancer mortality were substantially lower for gastrointestinal (13% RCTs, 42% deaths), gynecologic (3% RCTs, 8% deaths), and head and neck (3% RCTs, 6% deaths) cancers. There was a general alignment between the proportion of RCTs and deaths for lung, urologic, and brain cancers.

**Table 1.** Characteristics of RCTs Conducted by HICs Published From 2014 to 2017 With vs Without LMIC and UMIC Participation

Variable	No. (%)			*P* value
	All HIC RCTs (N = 636)	Enrolled Patients from LMICs and UMICs		
		Yes (n = 186)	No (n = 450)	
Disease site				
Breast	115 (18)	38 (20)	77 (17)	.18
Lung	84 (13)	28 (15)	56 (12)	
Gastrointestinal	116 (18)	25 (13)	91 (20)	
Head and neck	24 (4)	6 (3)	18 (4)	
Hematologic	119 (19)	36 (19)	83 (18)	
Urologic	64 (10)	24 (13)	40 (9)	
Gynecologic	35 (6)	5 (3)	30 (7)	
Skin	32 (5)	10 (5)	22 (5)	
Brain	20 (3)	4 (2)	16 (4)	
Other	27 (4)	10 (5)	17 (4)	
Treatment intent^a^				
Palliative	411 (65)	147 (79)	264 (59)	<.001
Curative	61 (10)	5 (3)	56 (12)	
Neoadjuvant/adjuvant	162 (26)	34 (18)	128 (28)	
Experimental group treatment				
Systemic	556 (87)	180 (97)	376 (84)	<.001
Radiotherapy	34 (5)	4 (2)	30 (7)	
Surgery	15 (2)	1 (1)	14 (3)	
Combination^b^	26 (4)	1 (1)	25 (6)	
Other^c^	5 (1)	0	5 (1)	
Control group treatment				
Active therapy	525 (83)	145 (78)	380 (84)	<.001
Placebo	63 (10)	32 (17)	31 (7)	
Observation/BSC	48 (8)	9 (5)	39 (9)	
Primary end point				
OS	198 (31)	66 (36)	132 (29)	<.001
DFS/EFS/RFS	142 (22)	24 (13)	118 (26)	
PFS/TTF	213 (33)	74 (40)	139 (31)	
QOL/toxic effects	20 (3)	2 (1)	18 (4)	
RR	35 (6)	13 (7)	22 (5)	
Other	28 (4)	7 (4)	21 (5)	
Industry funding				
Yes	469 (74)	177 (95)	292 (65)	<.001
No	167 (26)	9 (5)	158 (35)	

Abbreviations: BSC, best supportive care; DFS, disease-free survival; EFS, event-free survival; HIC, high-income country; LMIC, lower middle–income country; OS, overall survival; PFS, progression-free survival; QOL, quality of life; RCT, randomized clinical trial; RFS, recurrence-free survival; RR, response rate; TTF, time to treatment failure; UMIC, upper middle–income country.

^a^ Two studies were missing treatment intent.

^b^ Combined experimental arms included systemic with radiotherapy (n = 22), systemic with surgical (n = 3), and surgery with radiotherapy (n = 1).

^c^ Other experimental interventions included hyperthermia plus radiotherapy (n = 2), photodynamic therapy (n = 1), stem cell transplant (n = 1), and tumor-treating field (n = 1).

**Table 2.** Ranking of Top Cancers in Lower and Upper Middle–Income Countries

Top cancers	Rate, %	
	RCTs	Mortality^a^
Gastrointestinal	13	42
Lung	15	19
Gynecologic	3	8
Breast	20	7
Urologic	13	7
Hematologic	19	7
Head and neck	3	6
Brain	2	3

Abbreviation: RCTs, randomized clinical trials.

^a^ Mortality rate as per GLOBOCAN 2020.^12^

Trials that enrolled patients in LMICs and UMICs were more likely to be palliative intent compared with RCTs that only enrolled in HICs (147 of 186 [79%] vs 264 of 448 [59%]; 2 did not provide treatment intent; *P* < .00). Trials enrolling in LMICs and UMICs were more likely to be supported by industry (177 of 186 [95%] vs 292 of 450 [65%]; *P* < .001).

A summary of the results of the evaluated RCTs is presented in Table 3. The median sample size of HIC-led RCTs enrolling patients from LMICs and UMICs was larger than trials enrolling patients in only HICs (580 [IQR, 415-918] vs 402 [IQR, 226-692]; *P* < .001). The RCTs enrolling patients from LMICs and UMICs were also more likely to meet their primary end point (102 of 170 [60%] vs 127 of 387 [33%]; *P* < .001). Effect size and magnitude of benefit of the positive trials were comparable between the 2 groups. Trials that involved LMIC and UMIC participants were published in higher impact journals compared with RCTs that only enrolled patients in HICs (median impact factor, 27 [IQR, 20-36] vs 14 [IQR, 6-26]; *P* < .001).

**Table 3.** Results of All Oncology Randomized Controlled Trials Published by High-Income Countries During 2014 to 2017 (N = 636)

Variable	All HIC RCTs (n = 636)	Enrolled Patients from LMICs and UMICs		*P* value
		Yes (n = 186)	No (n = 450)	
Sample size, median (IQR)	474 (262-743)	580 (415-918)	402 (226-692)	<.001
*P* < .05 for primary end point, No. (%)^a^				
Total No.	557 (88)	170 (91)	387 (86)	
Yes	229 (41)	102 (60)	127 (33)	<.001
No	328 (59)	68 (40)	260 (67)	
HR for positive superiority RCTs^b^	n = 195	n = 92	n = 103	
Median (IQR)	0.69 (0.64-0.75)	0.70 (0.67-0.75)	0.67 (0.60-0.74)	.02
ESMO-MCBS grade, No. (%)^c^	145	73	72	
Substantial benefit (A,B,4,5)	45 (31)	19 (26)	26 (36)	.19
Not substantial benefit (C,1,2,3)	100 (69)	54 (74)	46 (64)	
Impact factor, median (IQR)				
All	21 (7-34)	27 (20-36)	14 (6-26)	<.001
Positive trials (n = 286)	25 (10-48)	34 (22-53)	15 (6-36)	<.001
Negative trials (n = 350)	18 (6-26)	24 (18-27)	14 (6-25)	<.001

Abbreviations: ESMO-MCBS, European Society of Medical Oncology–Magnitude of Clinical Benefit Results Scale; HIC, high-income country; HR, hazard ratio; LMIC, lower middle–income countries; RCTs, randomized clinical trials; UMICs, upper middle–income countries.

^a^ There were 559 superiority trials, but 2 indicated not applicable.

^b^ There were 229 positive superiority trials, but 34 did not report the hazard ratio.

^c^ Only reported for 145 of 229 positive superiority trials. In curative settings, A and B indicate substantial magnitude of benefit and C grade indicates nonsubstantial benefit. In noncurative settings, grades 4 and 5 indicate substantial magnitude of benefit and grades 1, 2, and 3 indicate nonsubstantial magnitude of benefit.

### Participation in RCTs vs Research Bibliometric Output

The association between LMIC and UMIC country-level participation in oncology RCTs led by HICs and total cancer research bibliometric output is reported in Table 4 and eFigure 3 in the Supplement (all countries) and the Figure (showing participation of LMICs in >5% and UMICs in >20% of RCTs). Several LMICs are overrepresented in our cohort of RCTs based on proportional cancer research bibliometric output: Ukraine (46% of RCTs but 2% of cancer research bibliometric output), Philippines (27% RCTs, 1% output), and Georgia (8% RCTs, 0.2% output). Several UMICs are also overrepresented in the study cohort of RCTs, including Russia (64% RCTs, 2% output), Romania (34% RCTs, 2% output), Mexico (31% RCTs, 2% output), and South Africa (30% RCTs, 1% output). An inverse association was seen for China (31% RCTs, 69% output).

**Table 4.** Country-Level Participation in 2014-2017 Global RCTs vs Cancer Research Bibliometric Output During 2007 to 2017

Variable	No. (%)	
	Global RCTs	Cancer research bibliometric output, publications
LMICs		
Total No.	84	41 200
India	42 (50)	27 601 (67)
Ukraine	39 (46)	801 (2)
Philippines	23 (27)	384 (1)
Egypt	12 (14)	6262 (15)
Georgia	6 (7)	78 (0)
Indonesia	3 (4)	650 (2)
Pakistan	3 (4)	2481 (6)
Vietnam	3 (4)	469 (1)
El Salvador	2 (2)	45 (0)
Morocco	2 (2)	1067 (6)
Tunisia	1 (1)	1362 (3)
UMICs		
Total No.	181	215 120
Russia	115 (64)	4835 (2)
Brazil	94 (52)	15 272 (7)
Romania	62 (34)	3457 (2)
China	56 (31)	154 373 (72)
Mexico	56 (31)	4126 (2)
South Africa	54 (30)	2076 (1)
Turkey	53 (29)	16 496 (8)
Thailand	48 (27)	3396 (2)
Bulgaria	34 (19)	841 (0)
Peru	29 (16)	432 (0)
Colombia	25 (14)	1131 (1)
Serbia	21 (12)	2455 (1)
Malaysia	11 (6)	3541 (2)
Guatemala	10 (6)	128 (0)
Bosnia and Herzegovina	9 (5)	285 (0)
Lebanon	9 (5)	944 (0)
Belarus	7 (4)	297 (0)
Costa Rica	5 (3)	208 (0)
FYR Macedonia	4 (2)	109 (0)
Venezuela, RB	3 (2)	306 (0)
Ecuador	2 (1)	122 (0)
Hungary	2 (1)	3220 (1)
Slovak Republic	2 (1)	1385 (1)
Algeria	1 (1)	275 (0)
Argentina	1 (1)	2787 (1)
Dominican Republic	1 (1)	15 (0)

Abbreviations: LMICs, lower middle–income countries; RCTs, randomized clinical trials; UMICs, upper middle–income countries.

**Figure.**
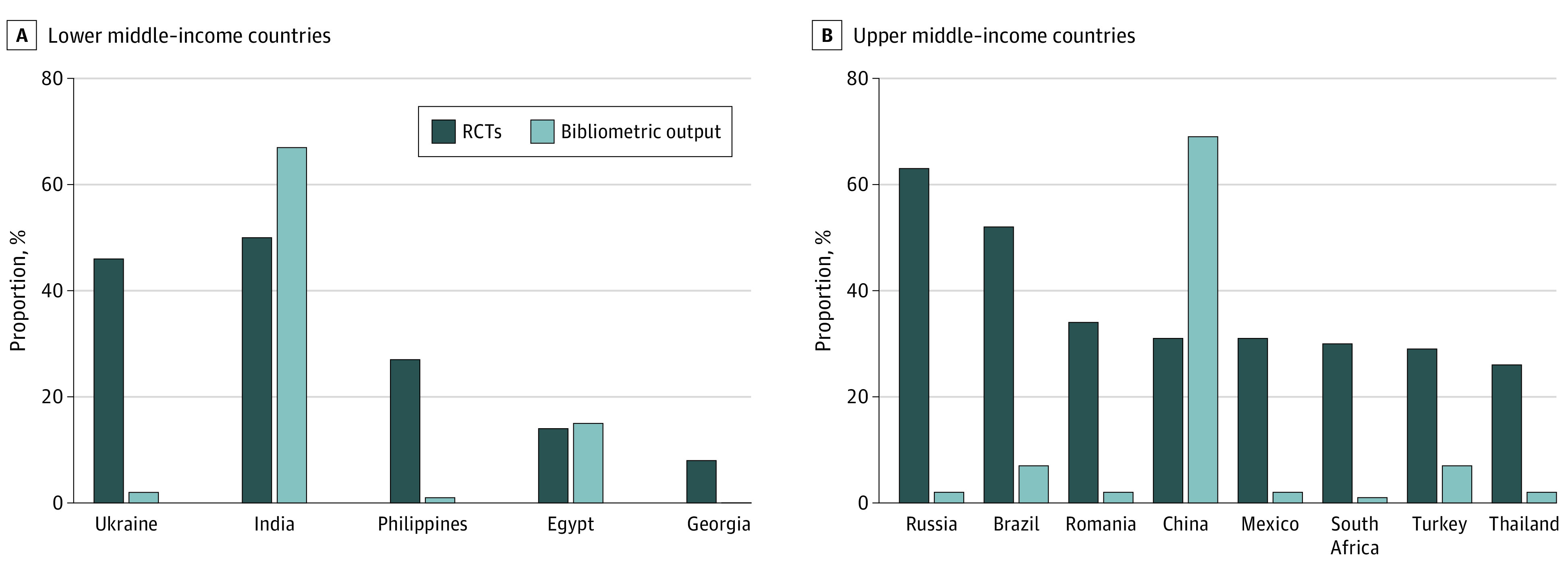
Country-Level Proportional Representation in Oncology RCTs Published From 2014 to 2017 Led by HICs for Selected LMICs and UMICs Shown Together With the Proportion of Total Bibliometric Output From 2007 to 2017 for Cancer Research Representation of lower middle–income countries (n = 84 trials) (A) and higher middle–income countries (n = 181 trials) (B). Countries included participated in at least 5% of lower middle–income countries trials and at least 20% of upper middle–income countries trials. HICs indicates high-income countries; LMICs, lower middle-income countries; RCTs, randomized clinical trials; UMICs, upper middle-income countries.

## Discussion

We have explored the involvement of LMICs and UMICs in global cancer RCTs. Several findings have emerged. First, only 8% of global oncology RCTs were led by investigators from LMICs and UMICs. Second, almost one-third of trials led by HICs enroll patients in LMICs and UMICs. Third, HIC-led RCTs that enroll in LMICs and UMICs do not match the burden of cancer in these countries. Fourth, HIC-led RCTs enrolling in LMICs and UMICs systematically differ from trials that only enroll in HICs; LMICs and UMICs are more likely to test new medicines in the palliative setting, have a larger sample size, and are more likely to be considered positive. Fifth, using the surrogate marker of cancer research ecosystem bibliometric output, the LMICs and UMICs most involved in HIC RCTs do not reflect strong endogenous cancer research ecosystems. Sixth, the impact of the Russian invasion of Ukraine highlights the fact that global RCTs are commonly conducted in fragile ecosystems. Until the war, Russia participated in 64% of global RCTs and Ukraine participated in 46% and were ranked 1 in UMIC and 2 in LMIC RCT.

There are numerous barriers to leading and developing research in LMICs and UMICs, including the overwhelming clinical workload, lack of research funding, and lack of research infrastructure. It is therefore not surprising that there are few RCTs led by LMICs and UMICs; most are led by HICs and funded by industry.^3,13^ Previous work has highlighted the lack of leadership roles provided to investigators from LMICs and UMICs; this lack of leadership role is consistent with one aspect of research parachutism whereby LMIC and UMIC investigators who are integral components of trial implementation are not granted authorship.^14^

Results from our primary study show that China and India account for 83% of the small number of RCTs led by UMICs and LMICs.^9^ In the present analysis we explored which countries participate in RCTs led by HICs. Unexpectedly, our data noted that the LMICs and UMICs most engaged in these trials are not those that have the strongest research ecosystems. This finding suggests that there is a policy disconnect: first, RCT participation is not building clinical research capacity and capability in these countries, and second, for LMICs and UMICs with strong research cultures, RCT participation is not a priority they seek or are offered. The 2022 invasion of Ukraine by Russia also brings into stark focus the fragile nature of many of the countries with patients participating in global RCTs.

There are several factors that make running RCTs in LMICs and UMICs appealing to investigators in HICs and industry including lower labor costs, fewer regulatory barriers, and a large pool of potential participants.^15,16^ We previously reported that RCTs led by UMICs and LMICs have smaller sample sizes.^9^ It was therefore an unexpected finding in the present analysis that RCTs led by HICs that enroll patients in UMICs and LMICs have larger sample sizes than trials conducted only in HICs. This difference may reflect the fact that trials powered to detect a small effect expand into UMICs and LMICs to boost accrual. Consistent with previous studies, we found that the cancers evaluated in these trials do not reflect the disease burden in these health systems.^17^

While the globalization of trials in oncology offers many benefits, there are potential risks. First, if a substantial proportion of patients are enrolled in LMICs and UMICs, the results may have limited generalizability to patients in HICs where these trials are often used to obtain regulatory approval. Internal validity may be limited if subsequent lines of therapy differ systematically between LMICs and UMICs vs HICs. Threats to the external validity in HICs may arise owing to pharmacogenomic differences in populations and other elements of care systems.^18,19^ At a deeper level there is debate as to whether RCTs conducted in LMICs and UMICs ultimately provide benefit to the broader population from which the research participants are drawn, and whether, more widely, such research contributes to a strong cancer research ecosystem.^6^ Given the high price of new cancer medicines, there are reasons to be concerned that new, effective treatments are unlikely to be available within the very health systems that contributed to the pivotal trial leading to regulatory approval.

The large financial stakes involved in cancer research and treatment pose both threats and opportunities for investigators and participants in low-resource settings. Participation in global RCTs can help LMICs and UMICs improve research infrastructure, strengthen clinical trial regulation, establish new collaborations, and provide research experience and expertise for investigators who may lack mentorship and methodologic training. However, the vast financial stakes introduce a power imbalance that should be carefully considered in light of possible exploitation of vulnerable populations, availability of the study drug after the trial, and other risks of research parachutism.^4,20^ Trial conduct and oversight mechanisms may also pose risks to participants in LMICs and UMICs. An analysis of 307 RCTs conducted in China and published in 2004 found that 90% of published trials in China did not report research ethics board review of the protocol.^21^

Our results offer policy-relevant insights into the cancer research ecosystem. One of the largest potential risks relates to the power imbalance introduced by financial support from the industry. It would be useful for health systems in LMICs and UMICs to make a greater investment in their domestic cancer research agenda. Clear regulation and governance are also needed to minimize the risks of industry financial support unduly influencing investigator decision-making and/or clinician delivery of care.^3^ It also may be beneficial for LMIC and UMIC governments to invest in building capacity and capability in clinical cancer research so investigators are better positioned to lead their own research that is in response to the needs of their own health system. There are many excellent global examples of where this is happening. In India, the National Cancer Grid has undertaken a major expansion in the national research agenda at both the cancer center level and by building research strength in depth through the Collaboration for Research Methods Development in Oncology model.^22,23^

The major role played in global oncology RCTs by Ukraine and Russia raises 2 fundamental issues. The first involves why Ukraine and Russia are overrepresented (ranked 2 in LMICs and 1 in UMICs) in the global portfolio of RCTs relative to their overall cancer research productivity. Points to be addressed are whether the overrepresentation reflects important efforts to build capacity, strong collaborative relationships between oncologists in Ukraine and Russia with oncologists in HICs, or opportunism by industry sponsors who are using money and power to gain access to a global population in health systems with low costs and fewer regulatory hurdles. The second key issue is whether the representation of the 2 countries will be influenced by the Russian invasion of Ukraine. These issues have not been addressed by the present study and will need to be considered by policy-makers and the broader oncology community. Delivery of health care (including cancer treatment) in Ukraine has been systematically degraded.^24^ For the foreseeable future, it will be challenging for patients in Ukraine to access routine cancer care let alone contribute to research. The inevitable reduction in clinical trial capacity within Ukraine and Russia may be offset by increased trial conduct in other UMICs and LMICs that have strong cancer research ecosystems, such as Brazil, China, Turkey, and India.

### Limitations

This study has limitations. First, we did not undertake a trial-level analysis of how many participants and investigators were involved in each trial; RCTs were broadly grouped as having LMIC and UMIC enrollment (yes/no). Second, our data do not allow us to comment on the details of trial implementation, governance, and funding in LMICs and UMICs. A future study will quantify investigator involvement at a more granular level. Third, for illustrative purposes we explored the proportion of RCTs in our cohort in which each LMIC and UMIC participated and compared it with country-level bibliometric research output. This is a crude approach to explore whether the countries that frequently participate in global RCTs are what one might expect based on other measures of cancer research system performance. Moreover, we did not expect a 1:1 ratio of proportions because many countries can join a single RCT. However, we noted some very high RCT/bibliometric output ratios that suggest outliers (eg, Ukraine ratio 23 and Russia ratio 31 compared with ratios in Brazil [7], India [0.7], and Egypt [0.9]). In addition, we grouped countries into economic regions but recognize that cancer burden and research capacity vary widely across countries, even within the same economic group.

## Conclusions

The findings of this cross-sectional study suggest that LMICs and UMICs contribute substantially to the global RCT ecosystem. Trials enrolling patients from low-resource settings are different from those conducted only in HIC settings. These data provide a signal that the trial design and cancers studied may not optimally match the needs of low-resource health systems. This discrepancy speaks to the urgent need for low-resource health systems to invest in domestic cancer research. Low and upper middle–income countries that participate in RCTs do not match overall cancer bibliometric output as a surrogate for research ecosystem strength. Reasons for this apparent discordance and how these data may inform future capacity-strengthening activities require further study.

## Notes

Supplement. **eFigure 1.** Conceptual Framework of Country-Level Participation in Global Cancer RCTs Published 2014-2017 With Country-Level Cancer Research Bibliometric Output (2007-2017) **eFigure 2.** Results of Search Strategy for All Oncology Randomized Clinical Trials Conducted Globally During 2014-2017 **eFigure 3.** Map Showing LMICs and UMICs Involved in HIC-Led RCTs
