# Omission in Title

**DOI:** 10.1001/jamanetworkopen.2022.35554

**Published:** 2022-09-20

In the Original Investigation titled “Participation of Lower and Upper Middle–Income Countries in Clinical Trials Led by High-Income Countries,”^1^ published August 18, 2022, a word was missing from the title. The word “Oncology” should have appeared before “Clinical.” The Supplement title has also been updated. This article has been corrected.^1^

## References

[1] Rubagumya F, Hopman WM, Gyawali B, . Participation of lower and upper middle–income countries in oncology clinical trials led by high-income countries. JAMA Netw Open. 2022;5(8):e2227252. doi:10.1001/jamanetworkopen.2022.27252 35980637 PMC9389348

