# Supplementary material for: Participation of Lower and Upper Middle–Income Countries in Clinical Trials Led by High-Income Countries
Source: JAMA Netw Open. 2022 Aug 18;5(8):e2227252. doi: 10.1001/jamanetworkopen.2022.27252 (PMC9389348; doi:10.1001/jamanetworkopen.2022.27252)
Supplement: Supplement. — eFigure 1. Conceptual Framework of Country-Level Participation in Global Cancer RCTs Published 2014-2017 With Country-Level Cancer Research Bibliometric Output (2007-2017) eFigure 2. Results of Search Strategy for All Oncology Randomized Clinical Trials Conducted Globally During 2014-2017 eFigure 3. Map Showing LMICs and UMICs Involved in HIC-Led RCTs [file jamanetwopen-e2227252-s001.pdf]

Supplementary Online Content

Rubagumya F, Hopman WM, Gyawali B, et al. Participation of lower and upper middle–income countries in clinical trials led by high-income countries. *JAMA Netw Open*. 2022;5(8):e2227252. doi:10.1001/jamanetworkopen.2022.27252

eFigure 1. Conceptual Framework of Country-Level Participation in Global Cancer RCTs Published 2014-2017 With Country-Level Cancer Research Bibliometric Output (2007-2017)

eFigure 2. Results of Search Strategy for All Oncology Randomized Clinical Trials Conducted Globally During 2014-2017

eFigure 3. Map Showing LMICs and UMICs Involved in HIC-Led RCTs

This supplementary material has been provided by the authors to give readers additional information about their work.

© 2022 Rubagumya F et al. *JAMA Network Open.*

eFigure 1. Conceptual framework of country-level participation in global cancer RCTs published 2014-2017 with country-level cancer research bibliometric output (2007-2017). In the example below, LMIC X participated in 55% of global RCTs but contributed only 5% of global cancer research output.

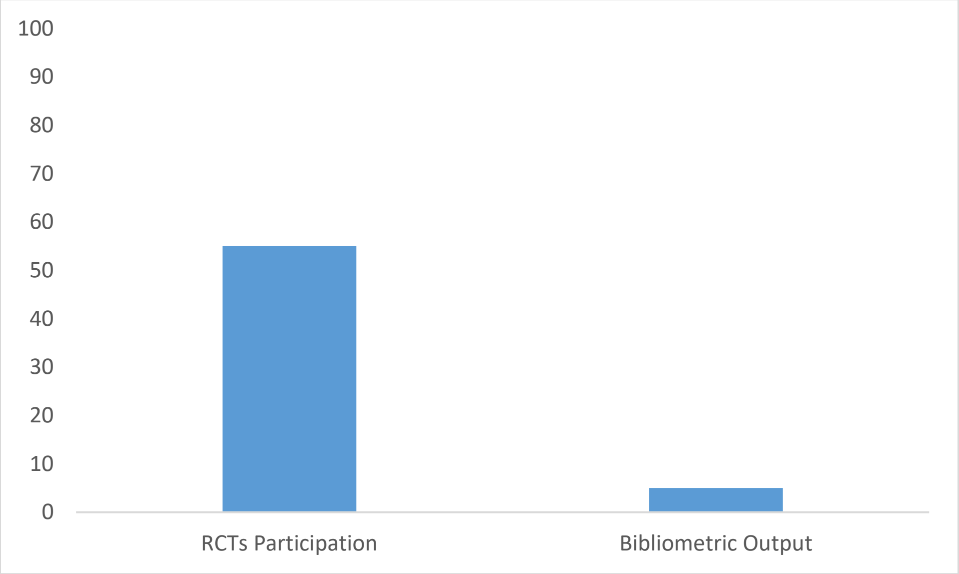

© 2022 Rubagumya F et al. *JAMA Network Open.*

eFigure 2. Results of search strategy for all oncology randomized clinical trials conducted globally during 2014-2017

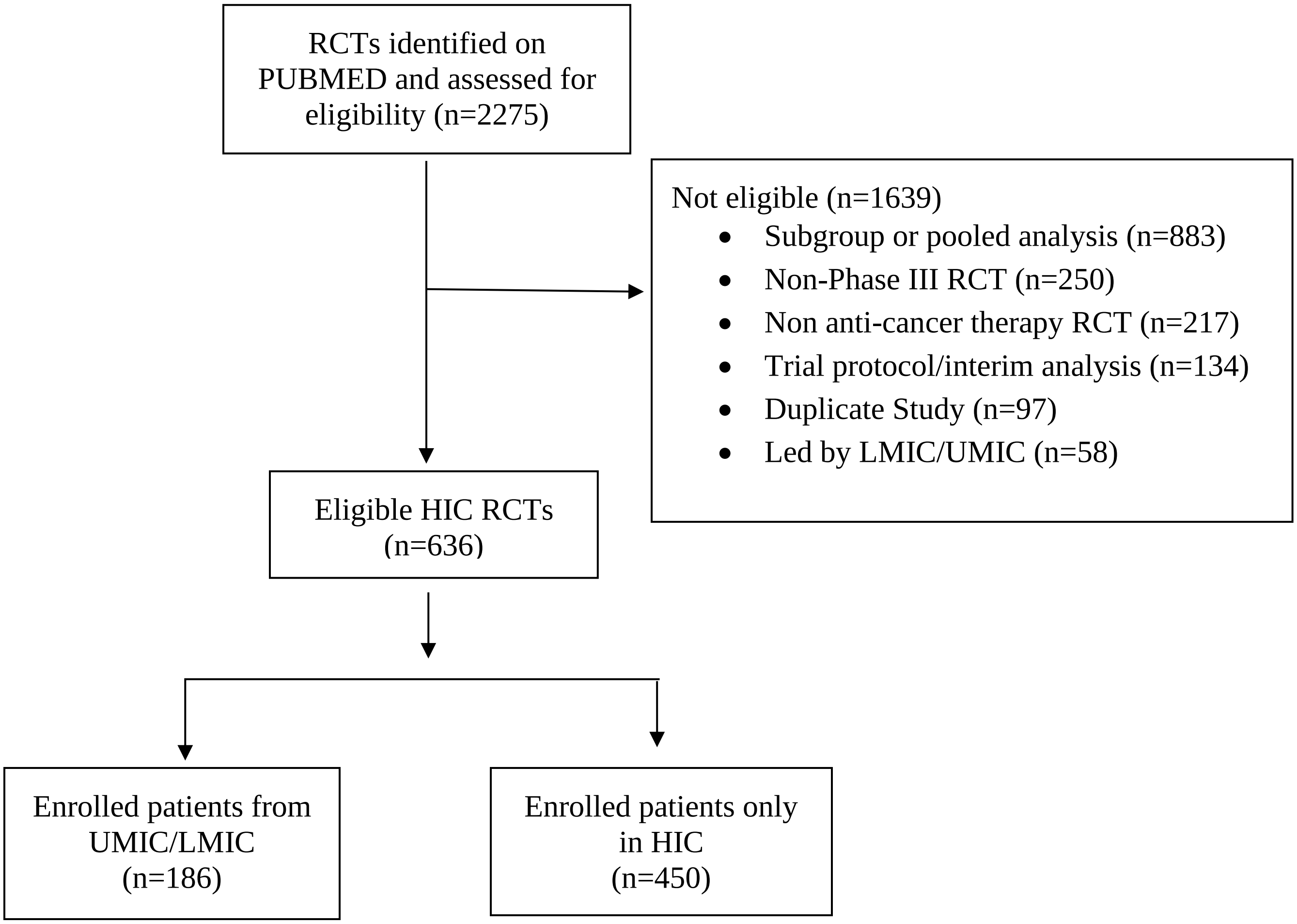

© 2022 Rubagumya F et al. *JAMA Network Open.*

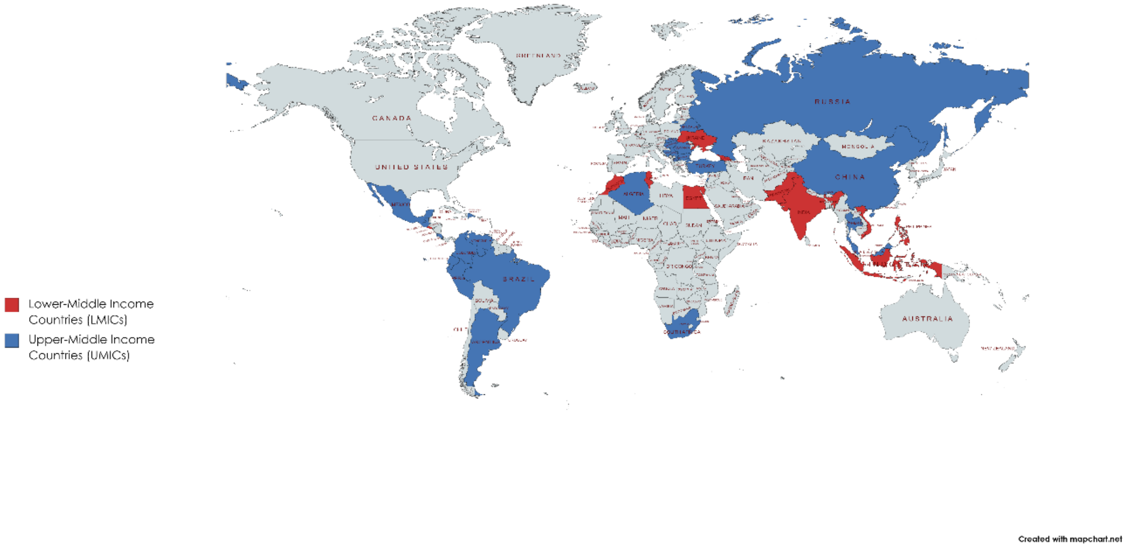

eFigure 3: Map showing LMICs and UMICs involved in HIC-led RCTs.

© 2022 Rubagumya F et al. *JAMA Network Open.*
